# Induction of substantial myocardial regeneration by an active fraction of the Chinese herb *Rosa laevigata Michx*

**DOI:** 10.1186/s12906-015-0795-0

**Published:** 2015-10-14

**Authors:** Huiyan Qu, Zhou Feng, Zhongyu Li, Censing Li, Mingfeng Tang, Zhen Zhou, Dianbin Li, Yongming Liu, Ming Li, Hua Zhou

**Affiliations:** Department of Cardiology, Shuguang Hospital, Shanghai University of Traditional Chinese Medicine, Shanghai, China; Laboratory of Innovative Medicine, Hong Kong, Hong Kong; Laboratory of Biotechnology, Dalian Institute of Chemical Physics, Chinese Academy of Sciences, 457 Zhongshan Road, Dalian, 116023 China; Queen Mary University of London, London, UK; Emergency Department, Tianshan Chinese Medicine Hospital, Changning District, Shanghai, China

**Keywords:** Myocardial infarction, Therapeutic cardiomyogenesis, Myocardium regeneration, Echocardiography

## Abstract

**Background:**

The replacement of lost cardiac tissues by regenerated myocardium would be a therapeutic ideal for myocardial infarction. The objective of this study was therefore to evaluate the ability of an active fraction that was isolated from *Rosa laevigata Michx* in therapeutic cardiomyogenesis in a myocardial infarction rat model.

**Methods:**

The myocardial infarction animal model was induced by the permanent ligation of the left anterior descending coronary artery in rats. The active fraction, which improves the survival rate and prevents ischemic reperfusion damage, was used to test the therapeutic effect of this fraction on myocardial infarction.

**Results:**

The oral administration of the active fraction for 4 weeks could progressively restore the decreased cardiac function due to myocardial infarction. The significantly improved cardiac function was probably attributed to the active fraction-induced myocardial regeneration, which replaced the lost cardiac tissues in the myocardial infarction animals.

**Conclusions:**

The property of this active fraction appears to be entirely novel and may provide a potential therapeutic alternative for myocardial infarction.

## Introduction

Pathologic stenosis or the occlusion of the coronary artery triggers a succession of events leading to cardiac ischemia or to the massive destruction of cardiac tissues in the distribution territory of the artery. Subsequent events of ventricle remodeling and consequent heart failure occur [1, 2]. Coronary heart disease (CHD) is the single largest killer of all diseases; more than one in five deaths are from CHD in Europe and in the US [3, 4]. Despite therapeutic advances, the replacement of the damaged cardiac tissues with regenerating myocardium remains a therapeutic ideal, as it is almost impossible for adult cardiomyocytes to considerably repopulate the pathologically lost myocardium through the inherited intrinsic cardiac repair mechanism [5–11].

Population-based clinical studies have demonstrated that although current advanced treatment modalities, including beta-adrenoceptor blockers, diuretics, and angiotensin-converting enzyme inhibitors, as well as surgical intervention procedures and mechanical assistance devices, are effective in improving symptoms and other outcomes in patients, these therapies restore neither the pathologic changes nor the functional performance of the damaged heart and have problematic complications [12, 13]. Heart transplantation may be an effective treatment for end-stage heart failure; the applicability of this procedure, however, is seriously limited by the availability of donor hearts and the concurrent immunorejection upon transplantation [14, 15]. Therefore, CHD remains the most prevalent cause of death, and the structural and functional restoration of the damaged heart remains a formidable challenge.

Research accomplishments during the last two decades on cardio-protective drugs or cell-transplantation-mediated therapies, such as stem cells or genetically modified cells, have constituted a revolution in regenerative medicine by creating living and functional tissues to replace damaged tissue or to restore organ function that is lost due to aging, disease and damage [16–20]. However, the reported severe or life-threatening complications that are associated with the use of exogenous stem cells/progenitors, including immunorejection, the risk of tumorigenicity and the insufficient efficiency of these progenitors/stem cells to acquire cardiac lineages to reconstitute the lost cardiac tissues, has hindered the clinical translation of this technology [21–23]. Therefore, an alternative strategy for cardiac regeneration and for the reconstitution of the damaged myocardium through an inherited cardiac repair mechanism is attractive because it may offer an ultimate solution to repair the damaged heart [6, 24].However, it was soon realized that the inherited cardiac repair mechanisms, including cardiogenesis through the proliferation and differentiation of resident cardiac stem cells (CSCs), the cardiogenic differentiation of bone-marrow-derived circulating stem cells that migrate to the site of damage, and the possible proliferation of neighboring cardiac myocytes under defined conditions, are in themselves insufficient to repopulate the lost myocardium or restore cardiac function [5, 6, 10, 11, 23]. Thus, it is highly desirable to develop new strategies that can potentiate the inherited cardiac repair mechanisms for the substantial repair of the damaged heart.

*Rosa laevigata Michx* (RLM) is mainly distributed throughout the southeast and southwest China, belongs to the rosaceae. RLM is a traditional Chinese medicine that is commonly used to treat pelvic inflammation, diarrhea, cough, asthma, and muscular, renal and hepatic damage [25–32]. Recent studies have reported that the oral administration of RLM extract obviously improved the survival rate and prevented ischemic reperfusion damage [33]. Further studies demonstrated that RLM significantly decreased DNA fragmentation, up-regulated the expression of Bcl-2, and down-regulated the expressions of p53 and active Caspase-3, −9 and −8 [33]. Thus, based on the properties of RLM in anti-oxidative stress, anti-inflammation, anti-apoptosis and especially the potential action for the treatment of ischemic stroke, we tested the potential of RLM for the treatment of ischemic myocardial infarction (MI). The aim of this study was to determine an alternative method that can substantially potentiate the inherited cardiac repair mechanisms for the regeneration of myocardium. The impact and prospects of this finding are important for clinical translation for the treatment of the diseased heart.

## Methods

### Preparation of the active fraction from *Rosa laevigata Michx* (aFRLM)

Bioassay-guided fractionation was performed to isolate the active fraction from the fruit of *Rosa laevigata Michx*. Briefly, the air-dried and powdered fruit of *RLM* (500 g) were collected from the Shanxi Province of China in September. The identity of the fruits of RLM by Prof. Ming Li, laboratory of Innovative Medicine Hong Kong. A voucher specimen (IM, JYZ-20130501) was deposited in the laboratory of Innovative Medicine Hong Kong. It was extracted with five volumes of absolute ethanol at room temperature for 1 day and ten volumes of 65 % ethanol for another 3 days. The extract was concentrated in a vacuum and yielded 40 g of crude ethanol extract. Reverse-phase column chromatography was used to isolate the active fraction from the extract using an Agilent 1200 series (Agilent corp. Ltd., USA). For HPLC, 50 mg of the extract was dissolved in 1 ml of methanol, and 20 μl of the sample was injected into the reverse-phase column (Waters, C18, 4.6 mm × 250 mm). The detection wavelength was set at 254 nm; 0.1 % acetic acid-H_2_O (solvent A) and methanol (solvent B) were used as the mobile phase, starting with 0 % B and increasing to 20 % B (10 min), 30 % B (10–40 min), 35 % B (40–50 min), 40 % B (50–60 min), 50 % B (60–70 min), 70 % B (70–80 min) and 100 % B (80–90 min) with a solvent flow rate of 1 ml/min at 25 °C.

### Preparation of bone marrow mesenchymal stem cells (MSCs) in vitro

Four-week-old male Sprague–Dawley (SD) rats were euthanized by CO_2_ inhalation in compliance with the American Veterinary Medical Association (AVMA) panel on euthanasia guidelines. The femora and tibiae bones were aseptically removed. The bone marrow cavity was flushed out under aseptic conditions with alpha IMDM culture medium [34, 35]. The bone marrow suspension was carefully agitated to obtain a single-cell suspension and centrifuged at 2000 rpm for 5 min. The resulting cell pellet was resuspended in 3 ml of ice-cold culture medium supplemented with 2 % PBS and 1 to 2 mM EDTA and was carefully applied on the top of the separation medium (Ficoll-Paque solution of 1.077 g/ml density) in a 50-ml centrifuge tube and centrifuged at 445 g for 30 min. The second layer containing mononucleated cells was carefully transferred to a tube and washed gently twice with PBS to remove Ficoll (1200 rpm for 5 min). The cell pellet was resuspended in ice-cold IMDM culture medium containing 10 % heat-inactivated FBS (GIBCO) and 1 % penicillin/streptomycin antibiotic mixture and cultured at 37 °C, 5 % CO_2_/95 % air in a humidified cell culture incubator for 24 h. The non-adherent cells in the culture were removed by washing the culture dishes with PBS, and the adherent cells were cultured by changing the culture medium every 3 days until the cells became nearly confluent after 10–15 days of culture.

### aFRLM-induced cardiogenic differentiation of MSCs in vitro

The obtained MSCs were cultured with the different fractions of the ethanol extract obtained from reverse-phase column chromatography. The cardiogenic activity of the fractions was compared to that of the crude extract. The fraction that was eluted from the column with 70 % methanol exhibited the best comparable activity compared to that of the crude extract in enhancing cardiogenic differentiation of cultured MSCs. Therefore, this fraction (aFRLM) was used in the following experiments.

The obtained MSCs were cultured with aFRLM (40 μg/ml IMDM culture medium) for 7–14 days. The expression of cardiac-specific myosin heavy chain (MHC) was assessed by immune cytochemistry at different time points [5, 11]. Briefly, 4 % paraformaldehyde in PBS was used to fix the cultured MSCs for 15 min, and the fixed cells were permeabilized with 0.5 % Triton X-100 for 15 min. Mouse monoclonal antibodies that were specific to MHC (1:600) (Santa Cruz) were used as the primary antibodies, and goat anti-mouse IgG antibodies that were conjugated with green Fluorophore (DyeMerTM 488/630 and 496/520) (Molecular Probe) were used as the secondary antibodies.

### Induction of the MI animal model and treatment protocol

All of the protocols conformed to the Guide for the Care and Use of Laboratory Animals as published by the U.S. National Institutes of Health and approved by the Animal Experimental Ethical Committee of the Zhangjiang High Technology Park. MI was induced in male Sprague–Dawley (SD) rats (200 ~ 250 g) by the permanent ligation of the left anterior descending (LAD) coronary artery as previously described [11]. In brief, the rats were anesthetized with an intraperitoneal Ketamine (100 mg/kg)/Xylaxine (10 mg/kg) mixture. The animals were mechanically ventilated with room air. The heart was exposed via a left anterolateral thoracotomy. The LAD was then ligated using a 7-0 polypropylene suture. The incision was closed in layers using resorbable 3-0 Vicryl sutures. The sham-operated rats received the same thoracotomy without LAD ligation.

After surgery, the rats were allowed to recover from anesthesia in a warm environment under continuous monitoring and care until they returned to consciousness. All of the experimental rats were postoperatively given antibiotics (benzathine penicillin G 150,000 U/kg and procaine penicillin G 150,000 U/kg) and analgesic (buprenorphine 0.03 mg/kg) every 8 h with frequent monitoring of the signs of pain/discomfort and decreased activities.

Three days after LAD ligation, the cardiac function was assessed using echocardiography. Only those rats exhibiting similarly decreased values of left ventricle ejection fraction (EF) and fraction shortening (FS) were included for further studies. The candidate animals were randomly divided into two groups. Eight rats in the test group were subject to aFRLM treatment (300 mg/kg weight/day in 2 ml of water) through gastric gavage daily for 4 weeks. Equivalent water was given orally to the animals of the vehicle-treated group (*n* = 8). All of the experimental animals were sacrificed after the last echocardiography measurements 4 weeks post-treatment using CO_2_ inhalation. Briefly, the animal-holding chamber was charged slowly with CO_2_ to a concentration of 70 % from a compressed CO_2_ gas cylinder. The gas flow was maintained for at least 1.5 min after apparent clinical death was verified.

### Echocardiography assessment of the therapeutic effect of aFRLM treatment

To assess the cardiac function of the experimental animals, echocardiography was performed in all of the experimental animals at different time points (prior to LAD ligation, 3 days post LAD ligation, 2 and 4 weeks after aFRLM treatment) under controlled anesthesia using a Toshiba Aplio XG Echocardiography with a PLT-1202 S linear array transducer [5, 36]. In each experimental subject, the M-mode tracing and 2-dimensional echocardiography of at least ten consecutive cardiac cycles were recorded from the parasternal long- and short-axis views. The left ventricle systolic function was assessed by calculating the EF and FS with the Modified-Simpson method using conventional echocardiography [37]. The left ventricular end systolic diameter (LVESD) and the left ventricular end diastolic diameter (LVEDD), as well as the systolic and diastolic wall thickness, were measured from the M-mode tracings using the leading-edge convention of the American Society of Echocardiography [5, 11, 36]. All of the data were analyzed offline with software that was installed in the ultrasound system. All of the measurements were averaged from ten consecutive cardiac cycles per experiment and were analyzed by an experienced investigator.

### Morphological and immunohistological assessment

The hearts of the sacrificed rats were removed and rinsed with PBS. All of the harvested specimens were sectioned for histological and immunohistochemical analyses. The left ventricle of these heart specimens were cut from apex to the base in two transverse slices and embedded in paraffin. The specimen paraffin blocks were sectioned respectively. The sections (5 ~ 20 μm) were stained with Masson’s trichrome according to the kit directions, which labels the nucleus black, the collagen blue and the myocardium red. The blue-stained fibrous scar areas were digitized and quantified morphometrically. The volume of the fibrous scar (≤ infarct volume) of a particular section was calculated based on the blue-stained area and the thickness of the section. The fibrous scar volumes of all of the sections were added to yield the total volume of the fibrous scar [5, 24]. To identify the regenerating myocardium, double immunohistochemical staining of the sections with cardiac-specific myosin heavy chain (MHC) and Ki-67-specific antibodies was performed according to previously described methods [5, 24]. Briefly, thin paraffin sections (5 μm) of the specimen were sequentially deparaffinized and stained with monoclonal rat-specific anti-Ki67 (Dako) and polyclonal anti-MHC antibodies (Santa Cruz). A specific secondary antibody that was conjugated with alkaline phosphatase (Santa Cruz) was used to visualize the positively stained cells [5, 11]. The newly regenerated myocytes were determined by counting the cells that were positively stained with both anti-Ki-67 and MHC concomitantly with the morphology of cardiac myocytes in each section.

### Statistics

All of the data, including cardiogenic differentiation in cell culture, morphometric analysis, and immunohistological analysis, were presented as mean ± SD. The statistical significance for the comparison between the two measurements from the two groups was determined using the unpaired two-tailed Student’s *t* test. Values of *P* < 0.05 are considered significant.

## Results

### Isolation of aFRLM from *Rosa laevigata Mich*

**Fig. 1:**
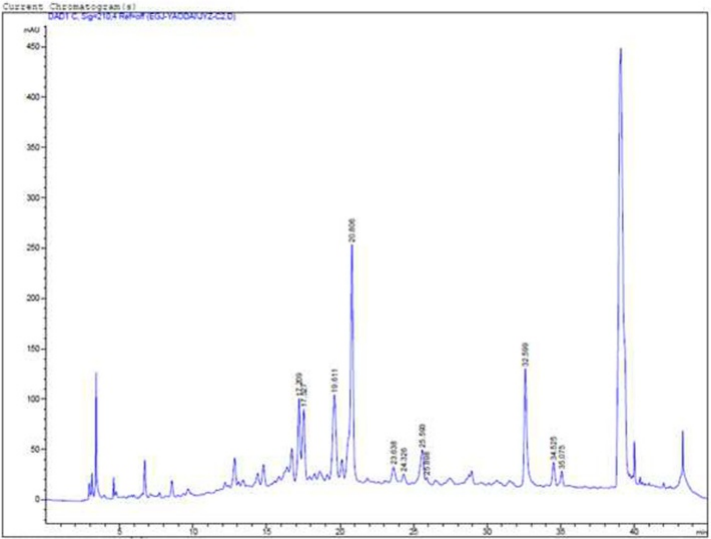
The fingerprint of the aFRLM

Bioassay-guided fractionation demonstrated that the fraction possessing the typical fingerprint as shown in Fig. 1 is the aFRLM. The structural analysis of aFRLM by nuclear magnetic resonance spectroscopic analysis demonstrated that aFRLM mainly contains the following compounds: laevigatins, potentillin, tormentic acid, 23-hydroxytormentic acid 28-O-β-D-glucopyranoside, 19α-hydroxyasiatic acid, and 19α-hydroxyasiatic acid-28-O-β-D-glucopyranoside.

### Induction of cardiogenic differentiation in MSCs

**Fig. 2:**
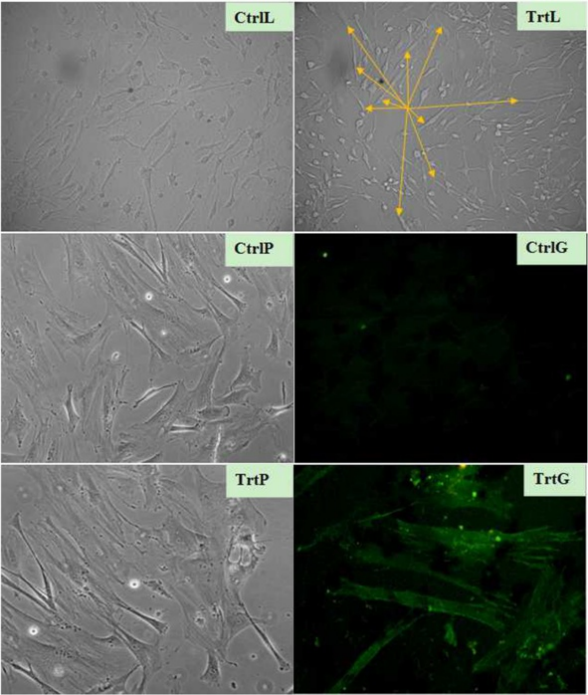
aFRLM-enhanced cardiogenic differentiation of cultured MSCs. The majority of the vehicle-treated MSCs (14 days) under low-power filed (CtrlL) and high-power filed (CtrlP) phase contrast microscope remained flat, irregular, asymmetrical and low-refractive with few MHC-positive (GFP) stained cells (CtrlG). In contrast, approximately 30 % of the cultured MSCs in the aFRLM-treated wells (14 days) under low-power filed (TrtL) and high-power filed (TrtP) became relatively elongated and had a high-refractive myocyte-like phenotype (*yellow arrowheads*) with approximately 30 % of these myocyte-like cells being positively (GFP) stained with MHC specific antibodies (TrtG)

The phenotype of bone-marrow-derived MSCs in anaphase appeared as flat, irregular, low-refractory, and polygon morphology. The MSCs of passages 5 to 6 were used to test the activity of aFRLM in the induction of cardiogenic differentiation. Our results demonstrated that approximately 20 % of the cultured MSCs in aFRLM-treated wells became elongated and had a refractive forming rod-like phenotype on day 7 compared to the flat, low-refractory, irregular, and asymmetry morphology in vehicle-treated MSCs. On day 14 of the treatment, approximately 30 % of the cultured MSCs showed the elongated myocyte-like phenotype, and more than 30 % of these myocyte-like cells were positively stained with MHC-specific antibodies (Fig. 2). In contrast, the majority of vehicle-treated MSCs remained flat, irregular, asymmetrical and low-refractive with few MHC positively stained cells at the same time point (Fig. 2) (*p* < 0.01). This result indicates the ability of aFRLM in promoting the cardiogenic differentiation of MSCs.

### Echocardiography assessment of heart function

**Fig. 3:**
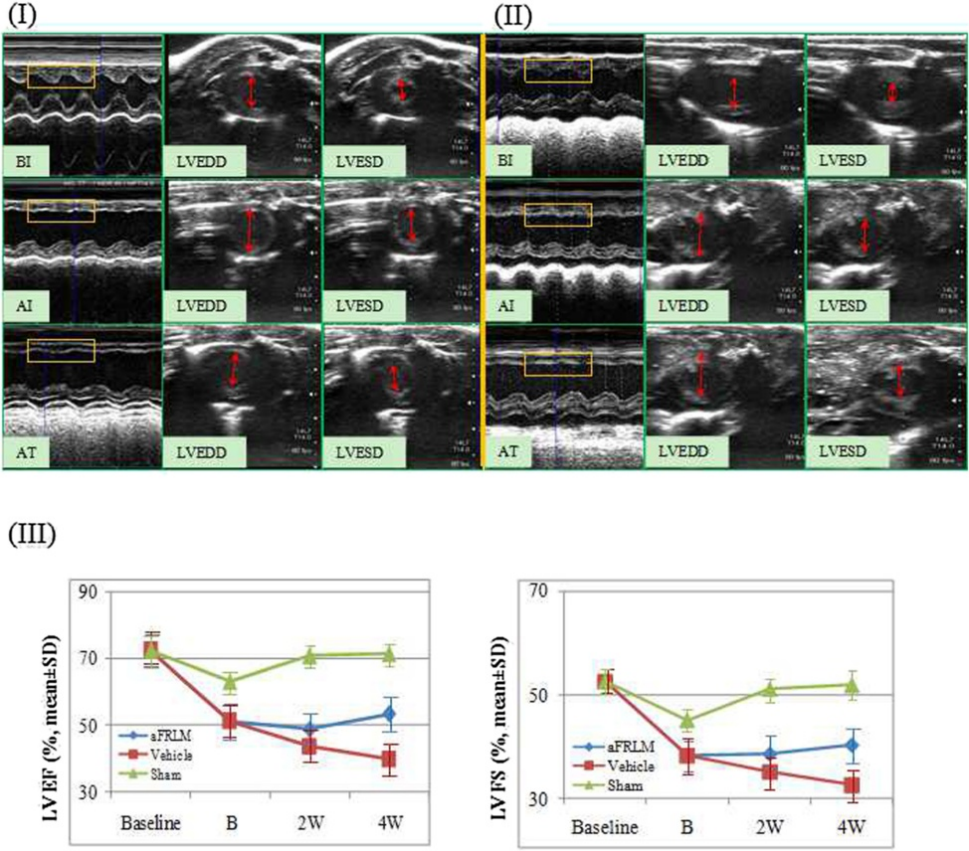
Echocardiography assessment of cardiac function. Representative M-mode and end diastolic/end systolic short-axis cross-sectional images of the left ventricle at the level of the papillary muscle of aFRLM-treated (I) and vehicle-treated (II) rat MI hearts. LVEDD: left ventricular end diastolic diameter; LVESD: left ventricular end systolic diameter; BI: normal rat echocardiography before MI induction; AI: images taken 3 days post infarction but prior to any treatment; and AT: images taken 4 weeks post either aFRLM or vehicle treatment. Before the LAD ligation surgery, the echocardiograms of the animals in both groups showed a normal motion of structure throughout several cycles in M-mode measurements of a short-axis view (I:BI & II:BI) (*yellow rectangular enclosed*) and normal LVEDD and LVESD (upper panels in I and II). Three days post ligation of LAD (I:AI & II:AI), anterior akinesis (*yellow rectangular enclosed*) and marked left ventricle dilation (*red arrowheads*) were observed in both groups of animals (middle panels in I & II). Four weeks of aFRLM treatment progressively improved the motion of the left ventricle anterior walls (*yellow rectangular*) and reduced the diastolic/systolic diameter (*red arrowheads*) (lower panel in I). In contrast, the vehicle-treated control hearts (II) remained in akinesis (*yellow rectangular*) and progressively increased in diastolic/systolic diameter (*red arrow heads*) (lower panel in II). III, LVEF and LVFS measurements demonstrated improved heart function with time in aFRLM treatment. Vehicle: vehicle-treated group; aFRLM: aFRLM-treated group; sham: open chest surgery without LAD ligation group; baseline: before ligation; B: 3 days post ligation; 2 W: 2 weeks post aFRLM or vehicle treatment; 4 W: 4 weeks post aFRLM or vehicle treatment. The graphs showed the restoration of the cardiac function in aFRLM-treated but not vehicle-treated hearts

The echocardiography assessment showed that the average baseline echocardiograms were approximately similar (73.3 ± 6.1 % for EF and 52.1 ± 5.1 % for FS) in all of the experimental animals before LAD ligation (Fig. 3). Three days post-LAD ligation prior to drug treatment, the average EF (58.4 ± 6.0 %) and FS (39.8 ± 4.8 %) values decreased by approximately 20 %. The decreased cardiac function was progressively worsened in the vehicle-treated group. In contrast, aFRLM treatment improved EF and FS by ~9.1 and 8.4 % (2 weeks) (*p* < 0.05) and by ~16.3 and ~15.2 % (4 weeks) (*p* < 0.01), respectively, compared to those of the vehicle-treated group (Fig. 3). The echocardiography of the sham-operated rats found that although the open chest surgery without LAD ligation caused the EF and FS to decrease slightly at 3 days post surgery, the slightly decreased heart function was fully restored at weeks 2 and 4 post surgery (Fig. 3). These results indicate that the oral administration of aFRLM improved the cardiac function of the MI rats.

### Quantitation of the infarct size

**Fig. 4:**
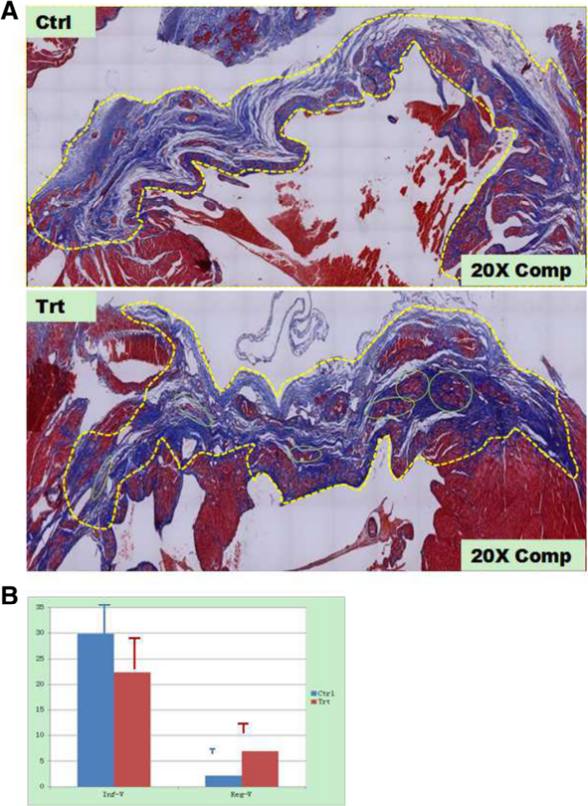
Morphometric assessment of the therapeutic effect of aFRLM on MI. **a**, The representative figures with the whole cross-field of infarction as stained by Masson’s trichrome method were composed of 130 (Trt) and 160 (Ctrl) consecutive microscopic photos (20×). The vehicle-treated MI heart (Ctrl) showed a blue-stained large and thinned infarct area (*yellow dashed line surrounding*). The fibrous scar of the infarct was stained blue. In contrast, the aFRLM-treated MI heart showed a smaller and less-thinned infarct area (*yellow dashed line surrounding*). More interestingly, many red-stained myocyte-like cell clusters (*green circles*) replaced the blue-stained fibrous scar and reduced the infarct volume in the aFRLM-treated MI heart. **b**: Inf-V, the infarct volumes in both aFRLM- (Trt) and vehicle- (Ctrl) treated hearts. Reg-V, the regenerating myocyte volumes in the hearts of both groups. Note, some newly formed cardiac myocyte clusters (**a**: *green circles*) replaced approximately one fourth (6.9 ± 2.3 %) of the original necrosed cardiac tissues, reducing the average infarct volume to 22.3 ± 7.0 % of the left ventricle volume in the aFRLM-treated group. In contrast, the average infarct volume occupied approximately 29.8 ± 7.3 % with less than 2.1 ± 1.1 % regenerating myocyte-like cells as observed in vehicle-treated hearts (*P* < 0.01)

The hearts of the sacrificed experimental animals were removed for histological, immunohistological and computed planimetric analyses. The territory of the ligated LAD appeared flattened and slackened with pale, uneven surfaces due to ischemic necrosis in vehicle-treated animals. In contrast, the corresponding territory in aFRLM-treated hearts showed a smaller area of such appearance with relative sound tension and thickness of the ventricle wall. The morphometric analysis of the infarct volume revealed that the original infarct volumes, as estimated as a percentage of the infarct volume of the total left ventricle volume, were approximately similar in both the aFRLM- and vehicle-treated hearts as estimated by the volume that the blue-stained regions occupied (Fig. 4a). However, after aFRLM treatment, some newly formed cardiac myocyte clusters were observed that replaced approximately one fourth of the original necrosed cardiac tissues, making the average infarct volume (22.3 ± 7.0 %) in aFRLM-treated group approximately one fourth smaller than that (29.8 ± 7.3 %) in the vehicle-treated hearts (*p* < 0.01) (Fig. 4b).

**Fig. 5:**
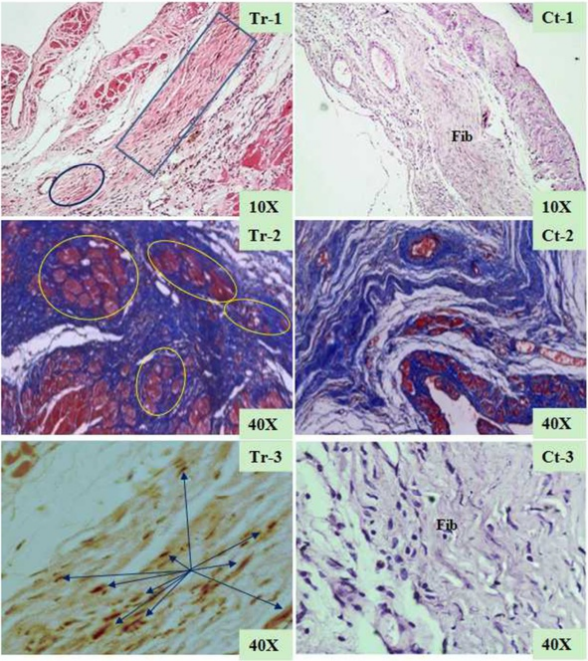
Morphological assessment of the therapeutic effect of aFRLM on MI. Representative micrographs of the aFRLM-treated (4 weeks) MI heart (Tr-1) showed that many red-stained myocyte-like cell clusters (*blue rectangular surrounding*) in the central area of the infarct were found that replaced the infarcted cardiac tissues. In contrast, the large area of fibrous scar was found throughout the entire infarct region (Fib) in the vehicle-treated MI heart (Ct-1) with few myocyte-like cell clusters. Masson’s trichrome staining demonstrated that although the infarcted cardiac tissues were replaced by blue-stained fibrous scar tissues, many red-stained myocyte-like cell clusters were found in the central infarct region (tr-2). However, in the vehicle-treated MI heart (Ct-2), the blue-stained fibrous scar replacement of the infarcted cardiac tissues was found throughout the entire infarcted region with few red-stained myocyte-like cells clusters. Some of the myocyte-like cells in the central infarct (*blue arrows*) were positively stained by both Ki-67 (brown nuclei)- and MHC (yellow cytoplasm)-specific antibodies in the aFRLM-treated MI hearts (Tr-3). Some of the regenerating myocytes joined together in tandem, forming a myocardium-like tissue. In contrast, fibrous scar replacement was found throughout the infarct region (Fib) with few myocyte-like cells that stained positively for both Ki-67 and MHC (Ct-3)

The ventricle wall within the scar area was not only smaller but also thicker in the aFRLM-treated animals than that in the vehicle-treated control rats (Fig. 4a). Many myocyte-like cell clusters with good alignment were found in the central infarct region, which substantially replaced the infarcted heart tissues and thus reduced the infarct volume by approximately one fourth of the total infarct volume upon 4 weeks of aFRLM treatment (Fig. 5). In contrast, the blue-stained fibrous scar was observed throughout the entire infarct region (Fig. 3a), and the red-stained myocyte-like cells, which were observed in clusters in aFRLM-treated hearts, were seldom found in the vehicle-treated MI heart (Fig. 5).

In order to determine whether these red stained myocyte-like cell clusters were newly regenerated cardiac myocytes, double immune-staining was performed using antibodies that were specific to MHC and Ki-67. These clustered myocyte-like cells, which were ~1/3 smaller than normal myocytes, were positively stained by both MHC- and Ki-67-specific antibodies (Fig. 5), indicating that these cells were newly regenerated cardiac myocytes. The longitudinal section through the infarct region of the heart specimen showed that some of the myocyte-like cells with colocalized Ki-67-positive nuclei, a MHC-positive cytoplasm and a striated phenotype were found in the infarct region. These results confirmed that these cells were newly regenerated myocytes replacing the infarcted heart tissues (Fig. 5).

## Discussion

First, this study demonstrated the effect of aFRLM in promoting cardiogenesis both *in vitro* and *in vivo*. Second, aFRLM-induced cardiogenesis was used to treat infarcted hearts in a sub-clinical MI animal model. The successful application of aFRLM for the treatment of MI would provide a convenient, safe and clinically well-accepted method for MI treatment.

To ensure reliability and to minimize the inaccuracies that derived from the variations of experimental animals due to the individual differences in the structure of the coronary arteriolar distribution and the system error of ligation surgery, all of the experimental rats received baseline echocardiography prior to LAD ligation and post LAD ligation, and only those rats exhibiting similar baseline values and similarly decreased values of LVEF and LVFS after ligation were included in further studies. To assess the therapeutic effect of aFRLM, the cardiac function was measured on a time-lapse basis during the treatment. To reduce the inaccuracies that derived from the variations in echocardiography measurement, the M-mode tracing and 2-dimensional echocardiography of at least ten consecutive cardiac cycles were recorded from the parasternal long- and short-axis views. For each M-mode measurement, at least six consecutive cardiac cycles were sampled [36]. All of the measurements were averaged over ten consecutive cardiac cycles per experiment and were analyzed by an experienced investigator.

To maximize the reliability of the measurement of the infarct volume of the heart specimen, the Masson’s trichrome blue-stained areas of the specimen were digitized and quantified morphometrically. The infarct size of a particular section was calculated based on the blue-stained area and the thickness of the section, and the total volume of the infarct was determined by adding the volumes of all of the sections within the infarct [5, 11, 24]. To identify the regenerating myocardium, we used Ki-67-positive staining to label the newly regenerated cells and MHC-positive staining to label cardiac myocytes. Therefore, counting the cells with the morphology of cardiac myocytes and the colocalization of both anti-Ki-67- and anti-MHC-positive stains in the infarct region are the criteria for regenerating myocytes. As a result, the reliability of the results that derived from the current tightly controlled experiment is considerably high. Because aFRLM showed its ability to enhance cardiogenic differentiation of MSCs *in vitro*, and the sizes of the newly regenerated cardiac myocytes in infarct region of aFRLM treated MI heart were approximately one-third smaller than those of the normal myocytes, the cellular origin of the regenerating myocytes are likely derived from circulating MSCs [38–40]. However, this assumption remains to be verified.

This study represents the first report that aFRLM may be used to repair of infarcted heart through enhancing myocyte regeneration. However, the underlying mechanism of how the oral administration of aFRLM in the MI model could significantly improve cardiac function and induce myocardial regeneration remains unknown. The occlusion of a major coronary artery, such as LAD, would cause a significant loss of functional cardiac myocytes through necrosis, intrinsic and extrinsic apoptosis pathways and autophagy. The molecular regulation of these cellular events is extremely complicated. For instance, the nuclear factor NF-κB super family of transcription factors has been implicated in the regulation of cell survival and inflammation in many cell types, including cardiac myocytes. Recent studies have demonstrated that NF-κB is cardioprotective during acute hypoxia and reperfusion injury. However, the prolonged activation of NF-κB appears to be detrimental and promotes heart failure by eliciting signals that trigger chronic inflammation through the enhanced elaboration of cytokines, including tumor necrosis factor, interleukin-1, and interleukin-6, leading to cell death. Further studies have indicated that the timing and duration of activation and the cellular context may mechanistically explain the differential outcomes of NF-κB signaling in the heart that may be essential for the future development of novel therapeutic interventions.

In our preliminary studies, we have tested the MSC culture with different concentrations (40, 80 and 160 μg/ml culture medium) of aFRLM. Among them, 80 and 160 μg/ml appeared cell toxic effect, 40 μg/ml showed the reasonable cardiogenic effect of MSCs. Therefore, the concentration of 40 μg/ml was used in this experiment. And we found that aFRLM treatment induced the transient up-regulation of NF-κB (12 h and 3 days reached the peak level), after which the NF-κB level decreased on day 7 and further decreased on day 14. Therefore, we hypothesize that the possible mechanism underlying the aFRLM-induced regeneration of myocytes is probably attributed to the multi-properties of aFRLM in anti-inflammation, anti-apoptosis, anti-oxidative stress, promoting cell survival and preventing ischemic reperfusion damage. As demonstrated in our preliminary studies, the transient up-regulated expression of NF-κB (12 h and 3 days post aFRLM treatment) may aid in the migration and accumulation of CSCs/MSCs to the infarct zone [41], and the ensuing down-regulation of NF-κB may contribute to the regulation of inflammation response (i.e., anti-inflammation, anti-apoptosis, and anti-oxidative stress), which would create a cardiogenesis-inductive environment for cardiogenic differentiation and for the maturation of the accumulated CSCs/MSCs [41–43].

The nature of aFRLM derived from the fruit of an edible plant and the convenience and safety of using aFRLM through oral administration demonstrated in this study make the possible immediate development of a new remedy for MI. In order to accelerate the development of aFRLM into a substantial clinical therapy, further studies are required, including investigations of the pharmacodynamic and pharmacokinetic properties and the metabolism of aFRLM *in vivo*. It is also of interest and of wilder clinical significance to investigate whether aFRLM is beneficial for old MI and ischemic cardiomyopathy. The demonstrated therapeutic effect of aFRLM in an animal MI model and the enhanced cardiogenic differentiation of MSCs *in vitro* by aFRLM suggest that aFRLM can be potentially developed into a specific drug for the effective treatment of MI. The property of aFRLM appears to be entirely novel and may provide a potential therapeutic alternative for myocardial infarction.

## Conclusion

In this study, we have identified an active fraction isolated from a Chinese herb *Rosa laevigata Michx* (aFRLM) that can enhance cardiogenic differentiation of cultured mesenchymal stem cells and induce substantial regeneration of myocardium. Our results demonstrated that oral administration of aFRLM to MI animals could significantly improve cardiac functional performance and induce myocardial regeneration replacing the necrosed cardiac tissues. The property of aFRLM appears to be entirely novel and may provide a potential therapeutic alternative for MI treatment.
